# A Rare Combination of Arterial and Venous Thrombosis as a Sequalae of COVID-19

**DOI:** 10.7759/cureus.32817

**Published:** 2022-12-22

**Authors:** Vugar Suleimanov, Kawther H Aljunibi, Batool M Almarhoon, Fatimah H Alhanabi, Hebah A Aldrazi

**Affiliations:** 1 Surgery, Jubail General Hospital, Jubail, SAU; 2 Medicine, Imam Abdulrahman Bin Faisal University, Al-Khobar, SAU

**Keywords:** acute limb ischemia, deep venous thrombosis, hypercoagulability, thromboembolism, covid-19

## Abstract

Coronavirus disease 2019 (COVID-19) has been a devastating condition claiming millions of lives, crippling countless people, and causing economic turmoil all over the world since the outbreak started in Wuhan Province of China in December 2019. Numerous papers have been published in the literature about COVID-19-related complications affecting almost all systems in the human body. One of the severe complications of this disease is thromboembolism, which affects both the arterial and venous systems and is well documented. There are few reports about both arterial and venous system involvement in the same patient. Herein, we report the case of COVID-19, who presented with critical limb ischemia caused by both arterial and venous thrombosis.

## Introduction

COVID-19 has been a hot topic and the focus of researchers worldwide since the start of the pandemic in 2019. According to the World Health Organization (WHO), COVID-19 is an infectious disease caused by a viral infection with severe acute respiratory syndrome coronavirus 2 (SARS-CoV-2). COVID-19 has been spreading due to its high transmissibility upon contact. Viral particles can be transmitted through small liquid particles, which range from larger respiratory droplets to smaller aerosols [1,2]. The presentation of COVID-19 can range from a mild respiratory illness that can be managed by supportive measures at home to a severe disease that requires admission to the intensive care unit (ICU). In addition, acute complications of COVID-19 include liver dysfunction, encephalopathy, cardiovascular events, acute respiratory distress syndrome, acute kidney injury, thrombosis, and many other critical conditions [3].

Thromboembolic events in COVID-19 were found to be pan-vascular. This is mainly attributed to the hypercoagulable state of COVID-19, endothelial injury after viral replication, and/or sepsis-induced coagulopathy. The resulting thrombus can be in the deep venous system, resulting in deep venous thrombosis (DVT), which may propagate and lead to pulmonary embolism. Moreover, thrombosis can also occur in the arterial system, resulting in acute arterial occlusion, stroke, or even myocardial infarction. This was also evident in post-mortem analysis, which showed a high prevalence of thrombosis in COVID-19 patients’ microvascular and macrovascular systems [4,5].

This case report presents a rare case of a thrombotic event induced by COVID-19 that affected both the arterial and venous systems in the same limb. The report aims to attract the attention of physicians to consider early evaluation of both the venous and arterial systems in COVID-19 patients, which may aid in early diagnosis and appropriate management of such cases, eventually reducing mortality and morbidity due to COVID-19.

## Case presentation

A 52-year-old male patient, newly diagnosed with type 2 diabetes mellitus (T2DM), presented to our hospital's emergency department (ED) with complaints of shortness of breath, a dry cough, and right foot pain lasting five days. The patient denied a history of fever or any other symptoms. The patient had never experienced similar pain in his limbs before.

**Figure 1:**
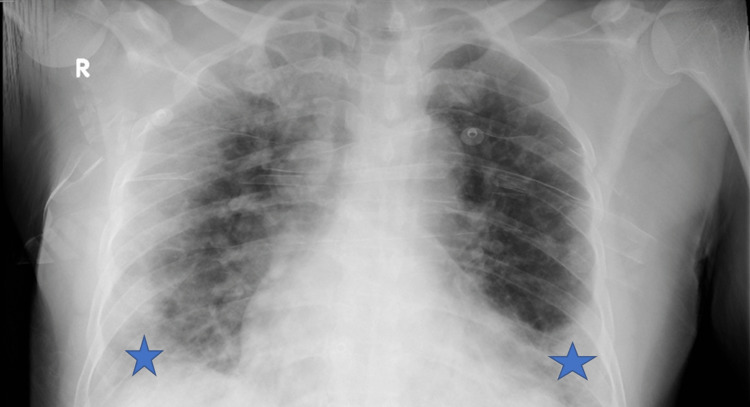
Chest X-ray shows COVID-19 related infiltrates in both lung fields and bilateral pleural effusion (Stars)

Upon physical examination, the patient was conscious, alert, ill-looking, and in distress due to pain. His vital signs on presentation showed a temperature of 37 °C, a heart rate of 119 beats per minute, a blood pressure of 153/82 mmHg, a respiratory rate of 30 breaths per minute, and oxygen (O_2_) saturation (SpO_2_) of 86% on room air. The patient was managed with supplemental oxygen through the non-rebreather mask at a rate of 15 L/min, and his SpO_2_ improved to 96%. Chest auscultation revealed scattered crackles. Right lower limb examination showed absent dorsalis pedis artery and posterior tibial artery (PTA) pulses, and the right foot was cold compared to the left. Chest X-ray findings (Figure 1) suggest COVID-19 pneumonitis, which was confirmed by a positive reverse transcription-polymerase chain reaction (RT-PCR) test.

Initial laboratory investigations revealed a white blood cell (WBC) count of 16.40 × 10^3^/µL, neutrophils of 86.2%, a D-dimer level of 1328 ng/mL, a prothrombin time (PT) of 13.80 seconds, an international normalized ratio (INR) of 1.21, and a partial thromboplastin time (aPTT) of 28.50 seconds.

**Figure 2:**
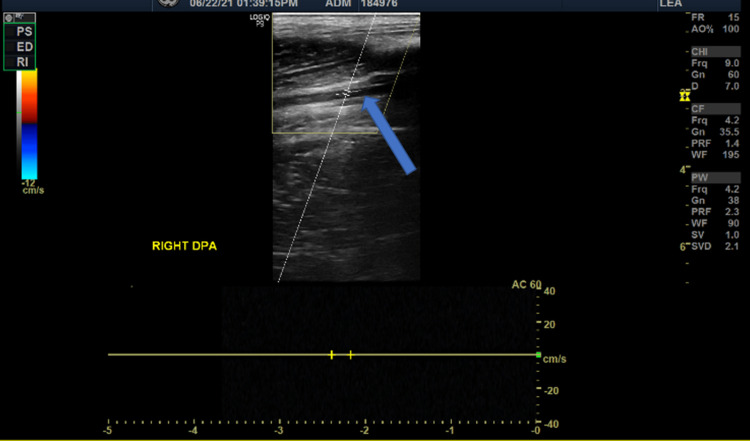
Doppler image shows absent blood flow in right dorsalis pedis artery (blue arrow)

**Figure 3:**
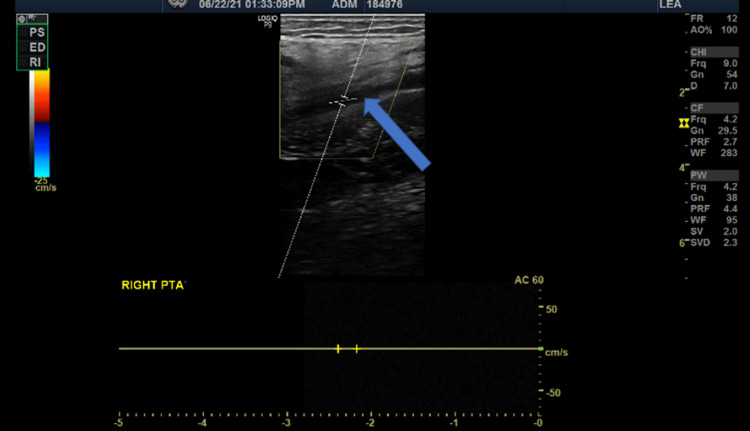
Doppler image shows absent blood flow in right posterior tibial artery (blue arrow)

**Figure 4:**
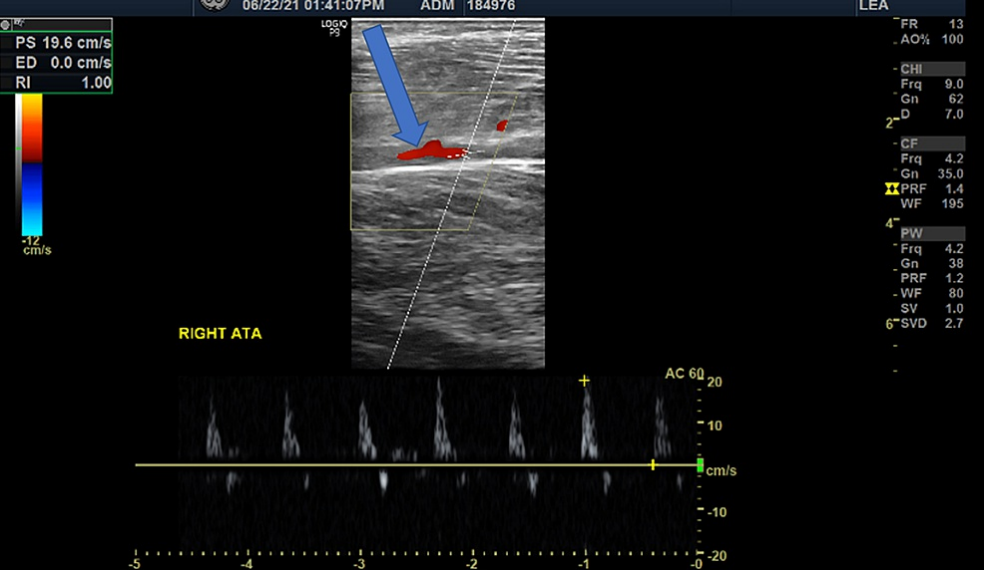
Doppler image of right anterior tibial artery shows presence of blood flow (blue arrow)

A lower limb duplex ultrasonography was performed, which revealed absent flow in the dorsalis pedis artery (DPA) (Figure 2) and posterior tibial artery (PTA) (Figure 3), while the anterior tibial artery (ATA) was patent (Figure 4).

**Figure 5:**
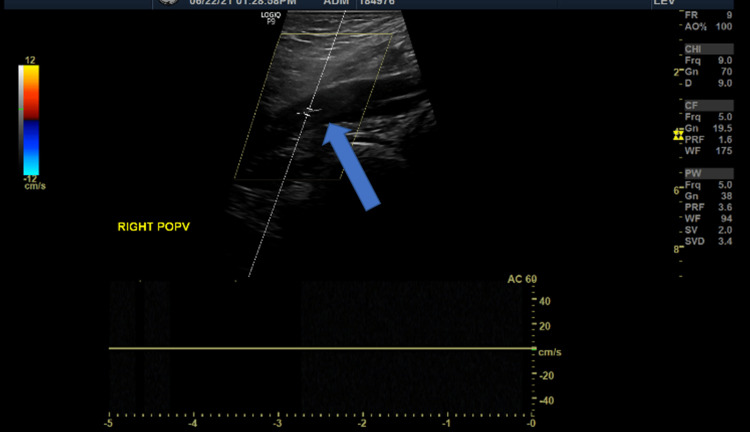
Doppler image shows non-compressible right popliteal vein with absent blood flow (blue arrow)

Furthermore, an incidental finding of hyperechogenicity within the popliteal vein denoting a thrombus was detected, suggesting DVT extending to the saphenopopliteal junction (Figure 5).

No source of embolism could be found. Electrocardiography (ECG) and echocardiography studies were normal. Since the patient had COVID-19 pneumonitis, we assumed he had COVID-19-related arterial and venous thrombosis of his right lower limb vessels.

The patient was admitted to the COVID-19 ward, and he was started on the management of his COVID-19 pneumonitis based on the available protocol according to the Ministry of Health in Saudi Arabia at that time. Regarding the patient’s limb ischemia, since the patient presented after five days of symptom onset, mottling had already started to appear in his right foot, and it was cold to touch; limb salvage was not considered a viable option. The management options were discussed with the patient, and amputation was offered, but he declined it initially. We decided to manage him with anticoagulation. At the time of admission, systemic anticoagulation with intravenous (IV) heparin was initiated. Later, it was changed to therapeutic subcutaneous (s/c) enoxaparin (1 mg/kg 12 hourly) injections. The patient did not improve, and the ischemia did not progress proximally either. The right foot remained cold up to the ankle, and mottling progressed. The patient continued to experience severe pain, which was difficult to control with narcotic analgesics. Chest findings started to improve; his O_2_ saturation on room air became 93%. The patient gave consent for amputation after repeated discussions and counseling.

After one week of admission, the patient was taken to the operating room (OR) after receiving prophylactic cefazolin 2 g IV. Transcutaneous oxygen saturation (tcPO_2_) was used to decide the level of amputation. At the ankle level, tcPO_2_ was 10 mmHg, 25 mmHg in the middle of the leg, and 30 mmHg in the upper third of the leg. Due to the fear of flap necrosis (low tcPO_2_ at the ankle and suboptimal tcPO_2_ at the middle of the leg), in addition to the presence of DVT in the same leg, the use of skew flaps instead of the usual Burgess flaps was chosen to cover the stump. A typical below-knee amputation was performed, where the bone was cut 12 cm below the tibial tuberosity. Blood clots in both veins and arteries were discovered during the surgery, which once again proved that the patient had arterial and venous thrombosis simultaneously. The wound was closed over the suction drain, and the patient was shifted to the ward. Routine wound care, supplemental oxygen, and anticoagulation were maintained, and the patient was discharged one week later in good condition. On a follow-up visit after one week, the wound was healed, the COVID-19 PCR test was negative, the chest X-ray findings had almost disappeared, and the patient’s O_2_ saturation on room air was 95%.

## Discussion

COVID-19 is a highly transmissible disease that was declared a worldwide pandemic by the WHO on March 11, 2020. The total number of cases of COVID-19 globally reached 642,924,560 as of December 8, 2022, in which the Europe region took the lead with 266,466,566 cases, followed by the Americas, Western Pacific, Southeast Asia, Eastern Mediterranean, and Africa, respectively. Locally, in Saudi Arabia, the total number of COVID-19 cases reached 826,000, of which the cumulative confirmed COVID-19 deaths per million people is 260.29 [6,7].

Globally, the increase in mortality and morbidity risks associated with COVID-19 is associated with advanced age, disability, and comorbidities, in addition to the low capacity of healthcare services. The high prevalence of chronic diseases in Saudi Arabia contributes to increased morbidity and mortality due to COVID-19. Additionally, there is insufficient data to determine the excess mortality rate caused by the COVID-19 pandemic compared to the years before it started in Saudi Arabia [8,9].

Several studies have been conducted to identify the possible complications of COVID-19 infection. A single-centered retrospective observational study in China found that end-organ damage was widespread among critically ill patients. The most common organs affected were the lungs, since acute respiratory distress syndrome was found in 67% of patients. The second commonest were liver dysfunction and acute kidney injury in 29% of patients, followed by cardiac injuries in 23% of patients [10].

In addition to the previously mentioned complications, coagulopathy, including DVT, and acute limb ischemia were commonly observed in many COVID-19 cases. DVT is defined as a blood clot formation in the deep venous system, more commonly in the lower limbs, while acute limb ischemia is defined as a decrease in limb perfusion that causes a potential threat to limb viability. There are two types of acute limb ischemia: critical and non-critical. Critical limb ischemia happens when the patient experiences the symptoms of rest pain, ulcers, and gangrene for more than two weeks. In patients with intermittent claudication, non-critical limb ischemia can range from asymptomatic to symptomatic [11,12].

In this case, COVID-19 presumably predisposed the patient to acute limb ischemia, which is supported by previous studies. According to Mansory et al., the incidence of venous thromboembolism was higher in critically ill patients diagnosed with COVID-19, reaching up to 24.1% [13]. Arterial occlusion, such as stroke, was recognized as one of the presenting clinical features of COVID-19, even in young individuals [14]. Furthermore, Kahlberg et al. examined 305 patients who presented during a COVID-19 emergency and discovered that COVID-19-positive patients had a higher rate of acute limb ischemia than non-COVID-19 patients (64% in COVID-19 patients compared to 23% in non-COVID-19 patients) [15].

Different risk factors were attributed to an increase in the incidence of arterial and venous thromboembolism among COVID-19 patients, including a very high D-dimer level, hospitalization, male gender, increased age, coronary artery disease, and a history of myocardial infarction. Consequently, increased mortality was observed after developing arterial and venous thromboembolism [16,17]. As evidenced by our case, multiple risk factors were found, such as male gender, increased age, and a high D-dimer level, that may have contributed to the development of deep venous thrombosis and acute limb ischemia.

COVID-19 patients' hospitalization in the ICU increases the risk of venous and arterial thromboembolism. A multi-centric study conducted in Saudi Arabia found that venous thromboembolism was detected in 1.8% of cases, with higher rates found in the ICU group compared to the non-ICU group. In contrast, arterial events were reported at 2.2% [17].

A population-based cohort study reported that the incidence of arterial complications declines more rapidly than venous complications after COVID-19 infection. However, the risk remains high within the first 49 weeks after the infection [18]. The late presentation of thrombotic events as a complication of COVID-19 was not the case for this patient. Signs and symptoms suggesting a thrombotic event were experienced by the patient within five days of clinical and radiological confirmation of the COVID-19 infection. The thrombotic events were proven in both the venous and arterial systems using doppler ultrasound. It showed an absence of blood flow in the right PTA and right DPA, indicating acute limb ischemia. Also, it showed a non-compressible right popliteal vein, suggesting DVT.

Depending on their severity, medical management is possible for DVT and limb ischemia. In the case of DVT, management depends on the extent and etiology. DVT can be divided into acute, short-term, and long-term phases. During these three phases, anticoagulation therapy is the primary treatment. More advanced procedures can be used in limb-threatening venous thrombosis, including catheter-directed thrombolysis, thrombectomy, and an inferior vena cava filter [11].

In the case of limb ischemia, Rutherford's criteria aid in the management. It depends on the presence or loss of sensory and motor functions and arterial and venous doppler findings. According to these criteria, limb ischemia is divided into different stages. The first stage indicates a normal leg. The second stage is divided into two categories: marginally threatened legs managed medically and immediately threatened legs managed surgically by revascularization. The third stage indicates a non-salvageable leg managed by amputation [12].

The combination of DVT and acute limb ischemia made it harder to depend only on medical management. However, the final decision on a typical below-knee amputation was made based on how badly the patient responded to the treatment and the progress of the disease. Initially, the patient was managed with anticoagulation since he refused the amputation option. Even though the severe pain and the coldness of his right leg, along with the presence of mottling, were indications of a non-salvageable leg from the beginning, the patient's informed choice was respected. However, the patient's condition did not improve, the mottling was progressing, and the pain became hard to control with strong analgesics such as narcotics. Eventually, amputation was inevitable.

The prognosis of acute limb ischemia is generally poor, mainly when associated with comorbidities such as COVID-19 pneumonitis. This is evidenced by a recent study conducted in 2020 to identify the prognostic features of acute limb ischemia in COVID-19 patients. It showed that abnormal coagulation profiles, such as significantly high D-dimers and fibrin degradation products, were more commonly associated with death in patients with COVID-19. Moreover, it has been suggested that the presence of both arterial and venous thrombosis is considered a poor prognostic factor compared to isolated venous thrombosis or isolated arterial thrombosis [19]. As demonstrated previously in the case, the patient had COVID-19 pneumonitis with an abnormal coagulation profile in addition to both arterial and venous thrombosis, which indicates the significance of early evaluation and management of such cases to avoid further progression and fatal consequences.

One of this case's limitations was not using imaging modalities other than duplex ultrasonography to confirm the diagnosis of limb ischemia. It is well known that digital subtraction angiography (DSA) is considered the gold standard diagnostic modality for limb ischemia. However, it should not be used as a replacement for duplex ultrasonography because it is invasive and carries a risk of complications, including injury to the vessels, kidney damage, and an allergic reaction to the contrast. Although computed tomography angiography (CTA) and magnetic resonance angiography (MRA) are highly sensitive and specific modalities, they require a long time to perform, which acute limb ischemia patients cannot tolerate. In contrast, there are advantages to the use of duplex ultrasound. It is a non-invasive, cheap, faster, and widely available imaging modality with no use of radiation. It is used to locate the obstruction and identify its degree [20].

## Conclusions

In such a rare combined sequela of COVID-19 that affects the arterial and venous systems in the same limb, treating physicians must know that this possibility could coincide. After that, early diagnosis and targeted therapy will be considered, and ultimately, a good prognosis is anticipated, including the chance of recovery and the disease outcome. In conclusion, ruling out DVT in COVID-19 patients presenting with critical limb ischemia is a prudent strategy, and patients must be counseled about the expected outcome, like the possibility of a higher-than-expected level of amputation, since DVT and arterial thrombosis in the same limb may contribute to impaired wound healing after the amputation. TcPO_2_ measurement at the time of surgery is a helpful tool in such situations to decide the level of amputation.
